# Supplementary material for: Polar Infection of Echovirus-30 Causes Differential Barrier Affection and Gene Regulation at the Blood–Cerebrospinal Fluid Barrier
Source: Int J Mol Sci. 2020 Aug 29;21(17):6268. doi: 10.3390/ijms21176268 (PMC7503638; doi:10.3390/ijms21176268)
Supplement: Supplementary file 1 [file ijms-21-06268-s001.pdf]

Supplementary Material

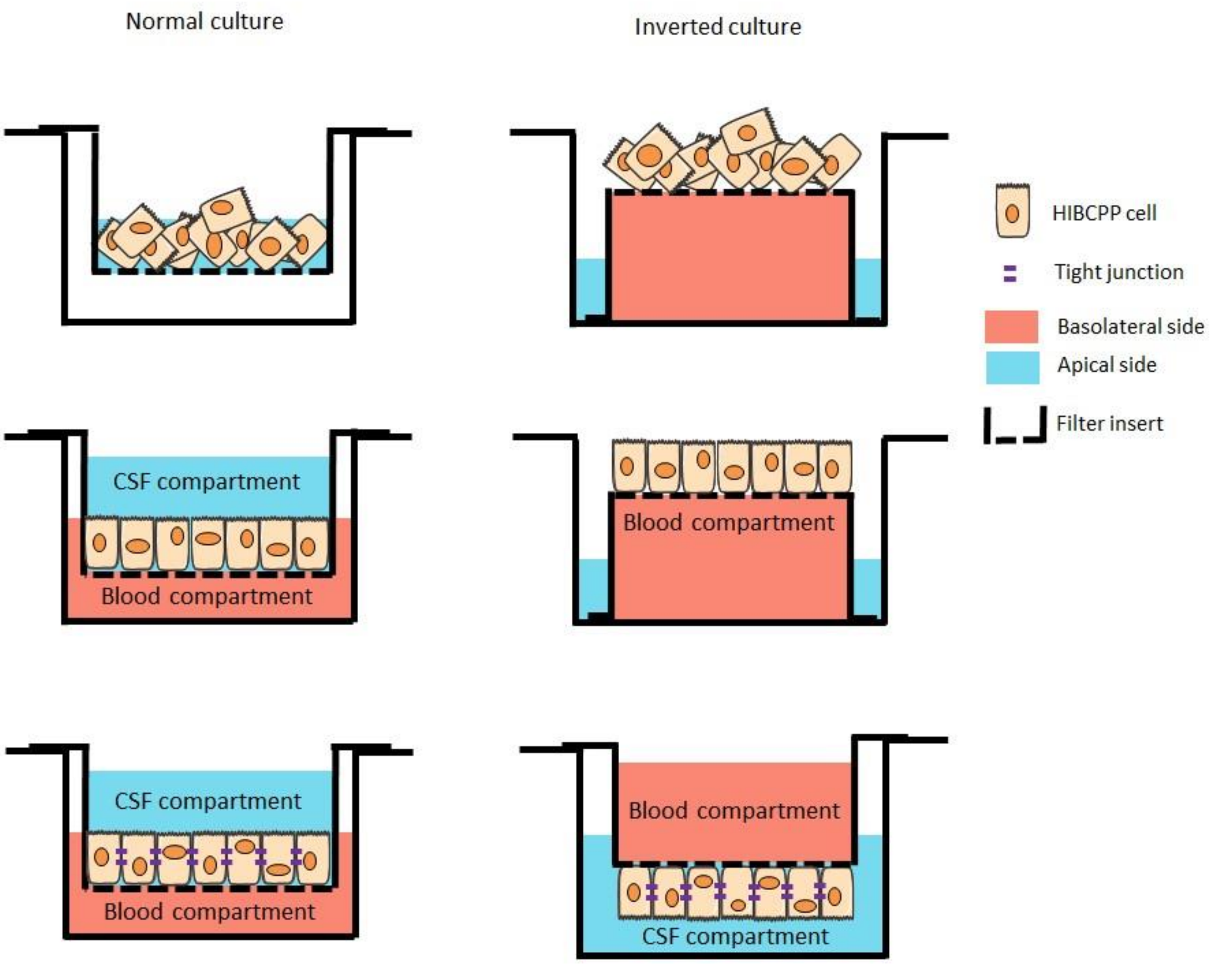

Supplemental figure 1. Schematic representation of normal versus inverted culture model of HIBCPP cells.

Schematic representation of HIBCPP cells in a normal culture (left) versus inverted culture (right). The normal culture leads to apical orientation of HIBCPP cells, whereas the inverted culture results in basolateral orientation of HIBCPP cells on the filter. Both systems lead to a high TEER and a low

paracellular dextran flux, which both correlate with the formation of tight junctions between adjacent HIBCPP cells.

gene	gene name	Log2FC	p-value
**ANKR7**	ankyrin repeat domain 37	-1,04	1,17E-07
**MIR29A**	microRNA 29a	-3,30	5,51E-05
**ZNF292**	zinc finger protein 292	-1,12	0,00015
**EDN2**	endothelin 2	-1,21	0,00021
**DUSP18**	dual specificity phosphatase 18	-1,10	0,00026
**HK2**	hexokinase 2	-1,05	0,00029
**CNGA1**	cyclic nucleotide gated channel alpha 1	-1,01	0,0016
**MIGA1**	mitoguardin 1	-1,04	0,0018
**ZNF74**	zinc finger protein 74	-1,17	0,0024
**MIR210HG**	MIR210 host gene	-1,19	0,0039
**GK**	glycerol kinase	-1,83	0,0048
**CBWD4P**	COBW domain containing 4 pseudogene	-1,10	0,0061
**LACTB2-AS1**	LACTB2 antisense RNA 1	-2,55	0,0065
**AC099791.2**	NA	-2,81	0,0066
**AL365181.2**	NA	-1,72	0,0074
**RASSF4**	Ras association domain family member 4	-1,48	0,0074
**DIP2C**	disco interacting protein 2 homolog C	-1,07	0,0080
**MYH10**	myosin heavy chain 10	-1,23	0,0086
**MIR222**	microRNA 222	-2,50	0,011
**FBXO24**	F-box protein 24	-2,69	0,011
**PPP1R3G**	protein phosphatase 1 regulatory subunit 3G	-1,04	0,012
**ALPL**	alkaline phosphatase, liver/bone/kidney	-1,73	0,012
**AL450998.2**	NA	-2,27	0,013
**PDE8B**	phosphodiesterase 8B	-2,12	0,015
**AL391244.3**	NA	-3,01	0,016
**AMT**	aminomethyltransferase	-1,21	0,017
**PLA2R1**	phospholipase A2 receptor 1	-1,11	0,017
**AC090673.1**	NA	-1,73	0,017
**KIAA2026**	KIAA2026	-1,01	0,017
**GLDN**	gliomedin	-2,56	0,019
**MIR221**	microRNA 221	-2,94	0,020
**TM6SF2**	transmembrane 6 superfamily member 2	-2,94	0,020
**ZNF658**	zinc finger protein 658	-3,69	0,021
**CASTOR1**	cytosolic arginine sensor for mTORC1 subunit 1	-1,41	0,023
**KCNJ14**	potassium voltage-gated channel subfamily J member 14	-1,30	0,023
**PGC**	progastricsin	-1,04	0,023
**NHLRC4**	NHL repeat containing 4	-2,94	0,023
**AC069503.1**	NA	-1,79	0,023
**LINC00910**	long intergenic non-protein coding RNA 910	-1,08	0,024
**TSNAXIP1**	translin associated factor X interacting protein 1	-1,48	0,024

**KNDC1**	kinase non-catalytic C-lobe domain containing 1	-1,22	0,026
**ROR1**	receptor tyrosine kinase like orphan receptor 1	-1,09	0,027
**AC004812.2**	NA	-1,69	0,028
**CATSPER1**	cation channel sperm associated 1	-1,53	0,028
**TMEM266**	transmembrane protein 266	-1,56	0,029
**ELFN1-AS1**	ELFN1 antisense RNA 1	-1,27	0,029
**AL139220.2**	NA	-1,95	0,031
**APLNR**	apelin receptor	-2,43	0,032
**ENPP1**	ectonucleotide pyrophosphatase/phosphodiesterase 1	-2,21	0,032
**PJVK**	pejvakin	-1,81	0,034

**genes**	**genes name**	**Log2FC**	**pvalue**
**IFIT3**	interferon induced protein with tetratricopeptide repeats 3	1,23	1,99E-32
**TXNIP**	thioredoxin interacting protein	1,46	3,03E-17
**RNA5-8SN1**	RNA, 5.8S ribosomal N1	1,03	2,14E-12
**CXCL3**	C-X-C motif chemokine ligand 3	1,48	8,64E-12
**GADD45B**	growth arrest and DNA damage inducible beta	1,46	3,41E-11
**RNA5-8SN4**	RNA, 5.8S ribosomal N4	1,04	8,45E-11
**RSAD2**	radical S-adenosyl methionine domain containing 2	1,88	1,08E-10
**IFIT1**	interferon induced protein with tetratricopeptide repeats 1	1,42	1,84E-10
**CLCF1**	cardiotrophin like cytokine factor 1	1,10	1,87E-10
**EDN1**	endothelin 1	1,07	2,18E-10
**IFIT2**	interferon induced protein with tetratricopeptide repeats 2	1,09	4,13E-10
**AC108134.2**	NA	2,13	2,39E-09
**IFNL2**	interferon lambda 2	4,22	4,14E-09
**CXCL2**	C-X-C motif chemokine ligand 2	1,11	4,22E-09
**IFNL3**	interferon lambda 3	4,04	1,75E-08
**IFNL1**	interferon lambda 1	2,81	1,70E-07
**SNORA80B**	small nucleolar RNA, H/ACA box 80B	4,08	7,50E-07
**MROH8**	maestro heat like repeat family member 8	1,99	1,48E-06
**NGFR**	nerve growth factor receptor	3,14	1,78E-06
**RNA5S6**	RNA, 5S ribosomal 6	4,60	3,50E-06
**RNA5S1**	RNA, 5S ribosomal 1	3,94	5,29E-06
**MIR3648-2**	microRNA 3648-2	3,56	6,25E-06
**RNA5S7**	RNA, 5S ribosomal 7	3,94	9,35E-06
**CYR61**	cysteine rich angiogenic inducer 61	1,05	1,06E-05
**AC002094.5**	NA	4,31	1,20E-05
**MT-CO1**	mitochondrially encoded cytochrome c oxidase I	1,53	2,42E-05
**RNA5S3**	RNA, 5S ribosomal 3	2,86	3,02E-05
**RNA5SP226**	RNA, 5S ribosomal pseudogene 226	2,00	4,76E-05
**MTCO1P12**	mitochondrially encoded cytochrome c oxidase I pseudogene12	1,79	4,78E-05
**MIR663AHG**	MIR663A host gene	3,63	6,31E-05
**RNA5S16**	RNA, 5S ribosomal 16	2,86	8,32E-05
**RNA5-8SN2**	RNA, 5.8S ribosomal N2	1,08	8,36E-05

**MIR3687-1**	microRNA 3687-1	4,12	9,30E-05
**RNA5SP145**	RNA, 5S ribosomal pseudogene 145	4,12	0,00011
**RNA5S4**	RNA, 5S ribosomal 4	2,31	0,00015
**MIR3648-1**	microRNA 3648-1	3,27	0,00016
**MT-TS1**	mitochondrially encoded tRNA serine 1 (UCN)	1,68	0,00023
**RN7SKP203**	RNA, 7SK small nuclear pseudogene 203	3,12	0,00025
**RNA5SP370**	RNA, 5S ribosomal pseudogene 370	3,90	0,00028
**AC008105.3**	NA	3,90	0,00034
**RNA5S13**	RNA, 5S ribosomal 13	1,96	0,00035
**RNA5S10**	RNA, 5S ribosomal 10	2,44	0,00038
**MTCO1P40**	mitochondrially encoded cytochrome c oxidase I pseudogene40	2,08	0,00051
**RNA5S5**	RNA, 5S ribosomal 5	2,35	0,00061
**RNA5S15**	RNA, 5S ribosomal 15	2,58	0,00069
**RNA5S2**	RNA, 5S ribosomal 2	2,46	0,00073
**MT-TD**	mitochondrially encoded tRNA aspartic acid	1,50	0,0013
**RGS16**	regulator of G protein signaling 16	2,17	0,0023
**CXCL10**	C-X-C motif chemokine ligand 10	1,18	0,0063
**CMTM3**	CKLF like MARVEL transmembrane domain containing 3	1,61	0,0067

Supplemental Table 1. Gene set enrichment analysis of basolateral infection with E-30 MOI 0,7 versus inverted control.

The tables show the p-value and log2-fold change (log2FC) for every differentially expressed gene for the 3 experiments. Genes with a p-value < 0.05, and a $|\text{log2FC}| > 1$ were considered as differentially expressed. Statistical analysis was performed using the R programing platform using DeSeq2 R/Bioconductor package. The blue table represents the first 50 down-regulated genes and the red table the first 50 up-regulated genes.

**gene**	**gene name**	**log2FC**	**p-value**
**ZNF837**	zinc finger protein 837	-2,00	0,0018
**EHD3**	EH domain containing 3	-1,71	0,0032
**AC020656.2**	NA	-1,65	0,0033
**LRRC27**	leucine rich repeat containing 27	-1,01	0,0045
**ADM2**	adrenomedullin 2	-1,94	0,0048
**INKA2**	inka box actin regulator 2	-1,54	0,0055
**PADI3**	peptidyl arginine deiminase 3	-1,95	0,0065

**DNAH1**	dynein axonemal heavy chain 1	-1,12	0,0080
**ZNF587**	zinc finger protein 587	-1,10	0,0088
**AMACR**	alpha-methylacyl-CoA racemase	-1,07	0,0091
**CDC42EP3**	CDC42 effector protein 3	-1,17	0,0094
**AC009414.2**	NA	-1,54	0,010
**AC078846.1**	NA	-1,51	0,010
**AC012073.1**	NA	-1,63	0,011
**PRR29**	proline rich 29	-1,86	0,013
**LGALS2**	galectin 2	-1,03	0,014
**U91328.1**	NA	-1,23	0,015
**IQCH-AS1**	IQCH antisense RNA 1	-1,34	0,022
**LINC01978**	long intergenic non-protein coding RNA 1978	-1,81	0,024
**C5orf63**	chromosome 5 open reading frame 63	-1,54	0,025
**ST3GAL3**	ST3 beta-galactoside alpha-2,3-sialyltransferase 3	-1,23	0,026
**ZNF782**	zinc finger protein 782	-2,06	0,026
**AC138696.2**	NA	-1,04	0,027
**HSPE1P6**	heat shock protein family E (Hsp10) member 1 pseudogene 6	-1,09	0,028
**PCK1**	phosphoenolpyruvate carboxykinase 1	-3,67	0,028
**SDCBP2-AS1**	SDCBP2 antisense RNA 1	-1,01	0,030
**AMT**	aminomethyltransferase	-1,06	0,030
**ANK1**	ankyrin 1	-1,25	0,031
**AC021504.1**	NA	-2,52	0,031
**AC009283.1**	NA	-1,55	0,032
**HIST1H2BN**	histone cluster 1 H2B family member n	-1,27	0,033
**E2F8**	E2F transcription factor 8	-1,61	0,034
**RPL19P21**	ribosomal protein L19 pseudogene 21	-1,24	0,034
**HCST**	hematopoietic cell signal transducer	-2,44	0,036
**SARDH**	sarcosine dehydrogenase	-2,86	0,037
**CCDC157**	coiled-coil domain containing 157	-1,22	0,038
**LTC4S**	leukotriene C4 synthase	-1,28	0,038
**AC016957.2**	NA	-2,11	0,039
**ZNF565**	zinc finger protein 565	-1,29	0,040
**SEPSECS-AS1**	SEPSECS antisense RNA 1 (head to head)	-1,01	0,041
**KCNK10**	potassium two pore domain channel subfamily K member 10	-1,12	0,042
**AL512637.1**	NA	-1,06	0,043
**ZBBX**	zinc finger B-box domain containing	-1,35	0,044
**SPDYE3**	speedy/RINGO cell cycle regulator family member E3	-1,13	0,044
**AGER**	advanced glycosylation end-product specific receptor	-1,44	0,044
**SPOCD1**	SPOC domain containing 1	-1,50	0,045
**AP001107.1**	NA	-1,10	0,047
**LINC01137**	long intergenic non-protein coding RNA 1137	-1,07	0,048
**ALG3P1**	ALG3, alpha-1,3- mannosyltransferase pseudogene 1	-1,20	0,048
**KHDC1**	KH domain containing 1	-1,02	0,048

gene	genes name	log2FC	pvalue
**TXNIP**	thioredoxin interacting protein	1,78	1,11E-115
**IFIT3**	interferon induced protein with tetratricopeptide repeats 3	1,91	1,81E-73
**OAS2**	2'-5'-oligoadenylate synthetase 2	1,42	1,20E-57
**CXCL3**	C-X-C motif chemokine ligand 3	2,17	9,28E-44
**IFIT2**	interferon induced protein with tetratricopeptide repeats 2	2,15	4,13E-41
**CXCL1**	C-X-C motif chemokine ligand 1	1,31	6,38E-35
**EDN1**	endothelin 1	1,85	3,30E-32
**CYR61**	cysteine rich angiogenic inducer 61	2,31	2,71E-28
**OASL**	2'-5'-oligoadenylate synthetase like	1,62	4,57E-25
**IFI44**	interferon induced protein 44	1,54	6,06E-25
**MX2**	MX dynamin like GTPase 2	1,72	2,44E-24
**CMPK2**	cytidine/uridine monophosphate kinase 2	1,94	2,61E-24
**PLK2**	polo like kinase 2	1,10	4,98E-24
**IFIT1**	interferon induced protein with tetratricopeptide repeats 1	2,12	2,27E-23
**IFNL2**	interferon lambda 2	5,80	3,04E-23
**NEURL3**	neuralized E3 ubiquitin protein ligase 3	1,03	1,82E-22
**IFI44L**	interferon induced protein 44 like	1,74	2,29E-22
**OAS1**	2'-5'-oligoadenylate synthetase 1	1,14	7,56E-22
**GADD45B**	growth arrest and DNA damage inducible beta	1,78	1,64E-21
**IFNL3**	interferon lambda 3	4,25	1,91E-21
**MX1**	MX dynamin like GTPase 1	1,56	2,09E-21
**ATF3**	activating transcription factor 3	1,26	6,36E-21
**RSAD2**	radical S-adenosyl methionine domain containing 2	2,84	1,59E-20
**G0S2**	G0/G1 switch 2	1,02	6,10E-19
**IFIH1**	interferon induced with helicase C domain 1	1,38	1,87E-17
**CLCF1**	cardiotrophin like cytokine factor 1	1,25	8,02E-16
**MYEOV**	myeloma overexpressed	1,12	1,13E-15
**JUN**	Jun proto-oncogene, AP-1 transcription factor subunit	1,26	2,08E-15
**DDX60**	DExD/H-box helicase 60	1,32	3,09E-15
**RND3**	Rho family GTPase 3	1,40	4,05E-15
**SLC2A3**	solute carrier family 2 member 3	1,01	4,54E-15
**ISG15**	ISG15 ubiquitin-like modifier	1,40	2,75E-14
**ARRDC3**	arrestin domain containing 3	1,23	4,35E-14
**PMAIP1**	phorbol-12-myristate-13-acetate-induced protein 1	1,37	9,71E-14
**SAMD9**	sterile alpha motif domain containing 9	1,56	3,42E-13
**FOS**	Fos proto-oncogene, AP-1 transcription factor subunit	1,26	5,09E-13
**ZC3HAV1**	zinc finger CCCH-type containing, antiviral 1	1,00	6,84E-13
**HERC5**	HECT and RLD domain containing E3 ubiquitin protein ligase 5	1,32	5,62E-11
**NGFR**	nerve growth factor receptor	3,36	3,47E-10
**CXCL2**	C-X-C motif chemokine ligand 2	1,09	7,42E-10
**EGR1**	early growth response 1	1,38	9,06E-10
**PIM1**	Pim-1 proto-oncogene, serine/threonine kinase	1,05	2,07E-09
**CXCL8**	C-X-C motif chemokine ligand 8	1,14	2,95E-09

**MROH8**	maestro heat like repeat family member 8	1,98	1,30E-08
**RNA5S1**	RNA, 5S ribosomal 1	4,37	2,84E-08
**IFIT5**	interferon induced protein with tetratricopeptide repeats 5	1,25	5,29E-08
**CXCL10**	C-X-C motif chemokine ligand 10	2,49	0,00019
**IFNL1**	interferon lambda 1	4,11	0,00091
**CCR1**	C-C motif chemokine receptor 1	1,05	0,0025
**IL6**	interleukin 6	3,23	0,0087

Supplemental Table 2. Gene set enrichment analysis of apical infection with E-30 MOI 20 versus standard control.

The tables show the p-value and log2-fold change (log2FC) for every differentially expressed gene for the 3 experiments. Genes with a p-value < 0.05, and a │log2FC│> 1 were considered as differentially expressed. Statistical analysis was performed using the R programing platform using DeSeq2 R/Bioconductor package. The blue table represents the first 50 down-regulated genes and the red table the first 50 up-regulated genes.

gene	gene name	Log2FC	p-value
**TMCC1**	transmembrane and coiled-coil domain family 1	-1,37	1,68E-20
**IFNL1**	interferon lambda 1	-1,94	3,39E-13
**ITGA5**	integrin subunit alpha 5	-1,25	7,81E-10
**EGR1**	early growth response 1	-1,40	1,45E-09
**CYR61**	cysteine rich angiogenic inducer 61	-1,13	6,94E-09
**IGFBP3**	insulin like growth factor binding protein 3	-1,04	1,08E-07
**ANKRD37**	ankyrin repeat domain 37	-1,15	1,81E-07
**IFNL3**	interferon lambda 3	-1,57	3,26E-07
**CXCL10**	C-X-C motif chemokine ligand 10	-1,45	4,76E-06
**IFNL2**	interferon lambda 2	-1,35	2,93E-05
**AKAP12**	A-kinase anchoring protein 12	-1,17	5,56E-05
**PTPRR**	protein tyrosine phosphatase, receptor type R	-1,13	0,00012
**AP001065.2**	NA	-3,52	0,00012
**HK2**	hexokinase 2	-1,08	0,00014
**MT1X**	metallothionein 1X	-1,23	0,00015
**ADM**	adrenomedullin	-1,29	0,00031
**PFKFB3**	6-phosphofructo-2-kinase/fructose-2,6-biphosphatase 3	-1,08	0,00053
**UPK1A-AS1**	UPK1A antisense RNA 1	-1,16	0,00057
**CCR1**	C-C motif chemokine receptor 1	-1,31	0,00061
**SLC2A3**	solute carrier family 2 member 3	-1,32	0,00071
**PFKFB4**	6-phosphofructo-2-kinase/fructose-2,6-biphosphatase 4	-1,41	0,00077
**ACHE**	acetylcholinesterase (Cartwright blood group)	-1,10	0,00094
**NDRG1**	N-myc downstream regulated 1	-1,17	0,0010
**ZNF292**	zinc finger protein 292	-1,02	0,0011
**PPP1R3G**	protein phosphatase 1 regulatory subunit 3G	-1,22	0,0014
**MIR221**	microRNA 221	-3,60	0,0016
**AC114803.1**	NA	-1,77	0,0017
**FTH1P15**	ferritin heavy chain 1 pseudogene 15	-1,78	0,0026
**GPR37L1**	G protein-coupled receptor 37 like 1	-1,27	0,0027
**ANGPTL4**	angiopoietin like 4	-1,13	0,0036
**LANCL3**	LanC like 3	-1,42	0,0038
**KRT16**	keratin 16	-1,25	0,0038
**HIVEP2**	human immunodeficiency virus type I enhancer binding protein 2	-1,18	0,0039
**ROCK1P1**	Rho associated coiled-coil containing protein kinase 1 pseudogene 1	-1,68	0,0052
**KRT17**	keratin 17	-1,09	0,0063
**FP565260.3**	NA	-1,06	0,0065
**A1CF**	APOBEC1 complementation factor	-1,46	0,0066
**CASTOR1**	cytosolic arginine sensor for mTORC1 subunit 1	-1,68	0,0069
**MIR29A**	microRNA 29a	-2,60	0,0077
**SLC27A3**	solute carrier family 27 member 3	-1,04	0,010
**B3GNT4**	UDP-GlcNAc:betaGal beta-1,3-N-acetylglucosaminyltransferase 4	-1,27	0,011
**TMEM40**	transmembrane protein 40	-1,39	0,011
**RP2**	RP2, ARL3 GTPase activating protein	-1,04	0,011

**YPEL1**	yippee like 1	-1,25	0,014
**PBRM1**	polybromo 1	-1,47	0,015
**GNG11**	G protein subunit gamma 11	-1,91	0,015
**LSM11**	LSM11, U7 small nuclear RNA associated	-1,09	0,016
**GK**	glycerol kinase	-1,65	0,016
**C1QTNF3**	C1q and TNF related 3	-2 ,16	0,017
**TRAF6**	TNF receptor associated factor 6	-1,18	0,026
**ADAM22**	ADAM metallopeptidase domain 22	-1,60	0,034

**genes**	**genes name**	**Log2FC**	**pvalue**
**TAF1C**	TATA-box binding protein associated factor, RNA polymerase I sub C	1,01	0,00018
**TAF10**	TATA-box binding protein associated factor 10	1,08	0,00021
**FCGR2A**	Fc fragment of IgG receptor IIa	1,64	0,0014
**ACTL10**	actin like 10	1,07	0,0015
**CDK20**	cyclin dependent kinase 20	1,52	0,0018
**AL158801.4**	NA	1,88	0,0024
**CDC42**	CDC42	1,18	0,0042
**ZNF837**	zinc finger protein 837	1,91	0,0042
**MT1G**	metallothionein 1G	2,25	0,0044
**AC106707.1**	NA	2,25	0,0045
**AC124248.1**	NA	2,08	0,0049
**MT-TD**	mitochondrially encoded tRNA aspartic acid	1,32	0,0054
**POLR2J4**	RNA polymerase II subunit J4, pseudogene	1,32	0,0062
**PIWIL4**	piwi like RNA-mediated gene silencing 4	1,18	0,0076
**MAP1A**	microtubule associated protein 1A	1,09	0,0079
**ANK1**	ankyrin 1	1,44	0,0081
**NRTN**	neurturin	1,00	0,0082
**MORN5**	MORN repeat containing 5	1,68	0,0083
**WDR38**	WD repeat domain 38	1,37	0,0092
**C5orf49**	chromosome 5 open reading frame 49	1,03	0,0092
**MT-TS2**	mitochondrially encoded tRNA serine 2 (AGU/C)	1,01	0,0093
**AC092171.3**	NA	1,57	0,011
**MIR148A**	microRNA 148a	1,55	0,011
**LGALS7B**	galectin 7B	1,89	0,011
**CARMIL3**	capping protein regulator and myosin 1 linker 3	2,64	0,012
**FAM3D**	family with sequence similarity 3 member D	1,08	0,013
**AC020656.2**	NA	1,40	0,014
**ADAT1**	adenosine deaminase, tRNA specific 1	1,29	0,014
**RPL19P21**	ribosomal protein L19 pseudogene 21	1,36	0,015
**SPDYE3**	speedy/RINGO cell cycle regulator family member E3	1,29	0,015
**UFSP1**	UFM1 specific peptidase 1 (inactive)	1,34	0,015
**KIF17**	kinesin family member 17	1,20	0,016
**CD14**	CD14 molecule	2,30	0,016
**AC138696.2**	NA	1,09	0,018

**AQP-1**	aquaporin 1 (Colton blood group)	2,12	0,018
**SDCBP2-AS1**	SDCBP2 antisense RNA 1	1,08	0,018
**CORO-6**	coronin 6	2,12	0,019
**ACTR3C**	ARP3 actin related protein 3 homolog C	1,01	0,019
**HNRNPABP1**	heterogeneous nuclear ribonucleoprotein A/B pseudogene 1	2,64	0,020
**UBA7**	ubiquitin like modifier activating enzyme 7	1,14	0,020
**COLCA1**	colorectal cancer associated 1	1,25	0,022
**MORN1**	MORN repeat containing 1	1,04	0,023
**SARDH**	sarcosine dehydrogenase	2,98	0,024
**C5orf63**	chromosome 5 open reading frame 63	1,52	0,024
**AC027281.2**	NA	2,52	0,025
**SLC47A1**	solute carrier family 47 member 1	1,04	0,027
**ZGLP1**	zinc finger, GATA-like protein 1	1,10	0,027
**ALG3P1**	ALG3, alpha-1,3- mannosyltransferase pseudogene 1	1,24	0,029
**E2F8**	E2F transcription factor 8	1,63	0,030
**CCDC126**	Coiled-coil domain containing 126	1,33	0,030

Supplemental Table 3. Gene set enrichment analysis of basolateral infection with E-30 MOI 0,7 versus apical infection with E-30 MOI 20.

The tables show the p-value and log2-fold change (log2FC) for every differentially expressed gene for the 3 experiments. Genes with a p-value < 0.05, and a │log2FC│> 1 were considered as differentially expressed. Statistical analysis was performed using the R programing platform using DeSeq2 R/Bioconductor package. The blue table represents the first 50 down-regulated genes and the red table the first 50 up-regulated genes.

Basolateral infection vs control		
Gene name	2-(-ΔΔCT)	S.D
INFλ-1	5,06	1,87
INFλ-2	39,48	24,09
IFIT-2	3,04	1,23
EDN2	0,91	0,10
ITGα5	2,19	1,00
CXCL8	1,94	1,01
CXCL2	2,41	0,40
CXCL3	5,18	1,17
IL-6	1,73	0,79

Supplemental Table 4. Fold change of *CXCL2, 3, 8, IL-6, IFNλ1, IFNλ2, ITGα5, EDN2* and *IFIT-2* genes following basolateral infection of HIBCPP cells with E-30 MOI 0.7 for 24 h determined by q-PCR.

Fold changes of basolaterally infected HIBCPP cells with E-30 in relation to uninfected control. The fold change for the qPCR data was calculated via the 2-$\Delta\Delta C_T$ method using GAPDH as an internal control, and the relative fold change was determined between basolaterally infected versus uninfected controls.

Apical infection vs control		
Gene name	2-(-ΔΔCT)	S.D
INFλ-1	3,52	2,10
INFλ-2	12,67	0,37
IFIT-2	4,72	1,72
EDN2	0,51	0,32
ITGα5	0,80	0,22
CXCL8	9,54	4,76
CXCL2	6,02	2,03
CXCL3	5,81	0,60
IL-6	3,18	2,38

Supplemental Table 5. Fold change of *CXCL2, 3, 8, IL-6, IFNλ1, IFNλ2, ITGα5, EDN2* and *IFIT-2* genes following apical infection of HIBCPP cells with E-30 MOI 20 for 24 h determined by q-PCR.

Fold changes of apically infected HIBCPP cells with E-30 in relation to uninfected control. The fold change for the qPCR data was calculated via the 2-$\Delta\Delta C_T$ method using GAPDH as an internal control, and the relative fold change was determined between apically infected versus uninfected controls.
